# Iterative Development of a Mobile Phone App to Support Community Health Volunteers During Cervical Cancer Screening in Western Kenya: Qualitative Study

**DOI:** 10.2196/27501

**Published:** 2022-02-24

**Authors:** Jacob Stocks, Yujung Choi, Saduma Ibrahim, Megan Huchko

**Affiliations:** 1 Center for Global Reproductive Health Duke Global Health Institute Durham, NC United States; 2 Kenya Medical Research Institute Nairobi Kenya; 3 Department of Obstetrics and Gynecology Duke University School of Medicine Durham, NC United States

**Keywords:** mHealth, cervical cancer screening, Kenya, HPV testing, user-testing, community health volunteers, mobile phone

## Abstract

**Background:**

To achieve the World Health Organization targets for cervical cancer elimination, low- and middle-income countries will need to develop innovative strategies to provide human papillomavirus (HPV)–based screening at a population level. Although mobile health (mHealth) interventions may help realize these goals by filling gaps in electronic specimen tracking and patient education, effective implementation of mHealth interventions is dependent upon context-specific development that is acceptable and usable by the target population. Detailed feedback should be gathered at the design and development stages to yield final products that reflect the needs, desires, and capabilities of target users.

**Objective:**

The aim of this study is to develop an mHealth app (mSaada) to support HPV-based screening in partnership with community health volunteers (CHVs) and program planners in western Kenya.

**Methods:**

A team of student programmers developed a prototype to meet previously identified gaps in screening: patient education, protocol support, data capture, and specimen tracking. The prototype was iteratively developed through 2 waves of in-person working sessions with quantitative (survey) and qualitative (in-depth interview) feedback. Research staff engaged key stakeholders from both urban and rural locations and with varying levels of experience in delivering screening services. During the sessions, participants completed simulation exercises and role-play activities to become familiar with the platform. Once feedback was gathered and synthesized after each wave of in-person data collection, developers implemented changes to improve mSaada functionality.

**Results:**

A total of 18 CHVs and clinicians participated in the in-person sessions. Participants found mSaada useful, easy to use, and would meet the needs of CHVs to provide HPV-based cervical cancer screening (electronic data capture, client education resources, and specimen tracking). They provided key feedback to enhance user experience, workflow, and sustainability. Key changes included altering the appearance of the wireframes, adding translation in additional local languages, changing potentially insensitive figures, alphabetizing lengthy dropdown menus, adding clinically relevant logic checks when entering data, and incorporating the ability to make real time edits to client records. They also made recommendations for additional features that might enhance mSaada’s impact at the facility and health system levels, specifically the inclusion of a report-generating tool consistent with the Ministry of Health standards.

**Conclusions:**

Using a process of iterative feedback with key stakeholders and rapid response from developers, we have developed a mobile app ready for pilot testing in HPV-based screening programs led by CHVs.

## Introduction

### Background

According to projections from the World Health Organization (WHO), incident cases of and annual deaths due to cervical cancer could increase by 37% and 47% to 777,000 and 459,000, respectively, by 2040 [1]. The vast majority of this disease burden is observed in low- and middle-income countries in sub-Saharan Africa and Southeast Asia [1,2]. To reduce rates of morbidity and mortality due to cervical cancer, screening and vaccination programs that target human papillomavirus (HPV), the causative agent in almost all cervical cancers, must be able to reach the target population in a cost-effective and culturally appropriate manner [3]. Such programs are nascent in many low- and middle-income countries and, despite the availability of simplified protocols, face infrastructural and financial challenges [4,5]. Kenya’s Ministry of Health has developed national guidelines for cervical cancer screening, which align with the WHO-endorsed framework for HPV-based screening and treatment. Although this may drive greater access to services throughout the country, there are significant barriers to implementation [3]. One such barrier is the use of nonformally trained lay providers, such as community health volunteers (CHVs), to deliver care. Although these individuals can reduce the clinical load on a strained health system, they require adequate supervision and support to carry out these services.

Poor adherence to clinical guidelines has been cited as a challenge to using CHV-led programs and a risk of delivering substandard care. An initiative to use lay health providers in Siaya district, Kenya, showed poor adherence to clinical guidelines by trained community health workers [6]. Further training, on-the-job supervision, and technical support have been shown to increase the quality of service delivery. A study conducted in the Morogoro region of Tanzania found that lay health workers “value supervision and appreciate the sense of legitimacy that arises when supervisors visit them in the village” [7]. However, it is important that when supervision is offered, it occurs in a respectful, nonjudgmental way, or it could lead to decreased self-efficacy and lowered desire to complete assigned tasks. The provision of job aids, such as pamphlets, flipcharts, mobile apps, and handbooks, has been cited as effective at increasing adherence and may provide a means to avoid overly critical supervision that still yields the necessary support [6,8].

Mobile health (mHealth) has been defined by the WHO as “medical and public health practice supported by mobile devices, such as mobile phones, patient monitoring devices, personal digital assistants, and other wireless devices” [9]. mHealth approaches, which typically use information sharing, education, communication, or data collection strategies to meet specified goals [10], have been used to deliver service reminders, promote behavior change, enhance medication adherence, provide health education, and collect and store patient data [11-15]. There is a large body of evidence supporting the feasibility, acceptability, and usability of a variety of mHealth interventions that target a wide range of health conditions such as depression, diabetes, HIV/AIDS, and cancer [16-22]. Although many mHealth interventions are patient sided, there are a growing number of provider-sided apps that seek to support and improve the delivery of health services [10,16]. These novel approaches provide a platform for the effective delivery of evidence-based practices targeting a range of health outcomes in often unreached or hard-to-reach populations and can be used to support task shifting of health delivery to CHVs or other lay health workers [23].

Effective implementation of mHealth interventions is reliant upon context-specific development that is appropriate, useable by the target population, and reflective of mobile phone ownership and rates of use [24,25]. Such considerations must be incorporated in the design and development stages of mobile apps and other interventions to yield final products that reflect the needs, desires, and capabilities of target users [25]. Otherwise, poor development of mobile apps could result in low levels of usefulness and user uptake [26]. A recent study by Huchko et al [27] showed that CHVs delivering cervical cancer screening in community- and facility-based settings in western Kenya desired more protocol and decision support tools to effectively complete screening. In addition, the study identified a clear need for continued education regarding the cause, risk, transmission, and prevention of HPV [28-30]. On the basis of high reported mobile phone ownership within Kenya and past research documenting success with SMS text messaging–based delivery of screening results in western Kenya [31,32], the introduction of a mobile app–based intervention to address gaps in education and provider support appears feasible in this context.

### Study Objective

The aim of this study is to iteratively develop and refine the mSaada mobile app in consultation with key stakeholders before small-scale pilot testing in health facilities in western Kenya.

## Methods

### Study Setting

This 2-wave qualitative study sought perspectives on the functionality of and user experience with a newly developed mobile phone app. Data collection was conducted in Migori and Kisumu, Kenya. Both locations, one rural and one urban, offer cervical cancer screening programs within local health facilities and were being considered for government-supported implementation of HPV-based screening. We chose these locations based on the target end user of the app, CHVs, who are commonly employed in both places.

### App Platform

mSaada, meaning *support* in Swahili, is a counseling and decision support tool designed for use by CHVs. As an android-based mobile phone app developed by students as part of a computer science course at Duke University, mSaada was designed to address logistical and educational gaps in HPV-based cervical cancer screening in western Kenya. The app features were developed based on prior research in Migori and Kisumu counties [27,28], and through web-based communication with Kenyan key stakeholders, who provided feedback on the overall functionality, wireframes, and pilot app. As described in Table 1, the app includes four main features to guide the CHVs through the entire process of screening, including counseling and decision-making, answers to frequently asked questions, data collection, and specimen tracking. Feature headings were defined based on stakeholder feedback and use more common, abbreviated language (*Add New Client*, *Screening Info*, *Questions*, and *Search Client*).

**Table 1:** Description of mSaada mobile app features.

Feature heading and purpose		Components
**Add New Client**		
	Collect relevant client data during screening	A 34-item clinical questionnaire collecting demographic and health history information
	Link client data to laboratory specimens for tracking and results notification	Barcode scanner for linkage of client records with laboratory specimens
**Screening Info**		
	Aid in education and counseling of clients	Cervical Cancer Education Module
	Aid in explanation of self-collection steps of HPV^a^-based cervical cancer screening	Kenya Ministry of Health Cervical Cancer Prevention Protocol
	—^b^	Explanatory figures and diagrams
**Questions**		
	Support client education	A total of 65 questions and answers addressing myths and misperceptions of HPV and cervical cancer, grouped by relevant topics including screening, treatment, and transmission
**Search Client**		
	Access, review, and edit client records	Searchable client record database

^a^HPV: human papillomavirus.

^b^Component applies to both purposes.

### Study Sample

To gather input and perspectives regarding mSaada and its features, we recruited individuals in three categories: experts (n=6), end users (n=6), and lay persons (n=6). Expert study participants were individuals with direct experience providing cervical cancer-related services, such as clinicians or study staff working in related research. End user study participants were individuals who had worked or were currently working as CHVs and had performed cervical cancer screening using HPV testing via self-collection. Lay person study participants were individuals who had no formal training or experience in cervical cancer screening. Research staff identified potential study participants based on prior engagement with the individuals or by recommendation from partner facility staff. Study staff recruited participants by phone or in-person before the beginning of the study period.

### Study Design

The iterative development period lasted 8 weeks in total and consisted of alternating waves of data collection and integration of feedback into the mSaada platform. During data collection waves, participants attended day-long feedback sessions that were categorized by participant group (ie, experts, end users, and lay persons). To obtain a combination of novel and continuous feedback on mSaada, we asked 2 representative experts, end users, and lay persons (6/18, 33%) to participate in both waves of data collection activities, completing an in-depth interview during both waves. All other study participants (12/18, 67%) were asked to attend only 1 day of feedback sessions, completing only 1 in-depth interview. This resulted in 24 in-depth interviews among the 18 participants, with an average length of 45 minutes.

Feedback sessions (n=6) began with a description of study aims, intended methods of data collection, and completion of written informed consent. Following this discussion, researchers provided detailed, screen-by-screen explanations of the mSaada app and its features, allowing participants to follow along using study phones loaded with the platform during the demonstration. After initial hands-on familiarization in a group setting, participants completed simulation activities in pairs, including CHV and client role-plays and example scenarios to gain more experience with the platform [33]. Explanation, familiarization, and role-plays with mSaada lasted approximately 3 hours, on average. Thereafter, we conducted individual in-depth interviews to gather detailed feedback about the platform. Both the group exploration and familiarization sessions and individual in-depth interviews were conducted in English and audio-recorded for transcription. We completed transcription using Otter.ai, a free, open-source software. Study staff transcribed recordings using the software, and reviewed and edited resulting transcripts for accuracy. Researchers followed the same protocol for both waves of data collection. All study activities occurred within the Kisumu Office of the Duke Center for Global Reproductive Health and the Migori County Hospital. Group feedback sessions were conducted within the conference area of the office, whereas individual in-depth interviews were conducted within private rooms. Research staff and app developers convened to discuss and integrate key participant feedback after it was gathered.

### Communication With App Developers

Researchers consolidated data from the first wave of interviews into a master list of recommended changes and updates. The resulting 12-page document was shared with Kisumu- and Durham-based researchers to focus and prioritize revision efforts. Although most of the recommended changes were considered for integration before the second wave of interviews, some participants suggested changes that were not feasible to undertake between the 2 feedback waves. We addressed these changes, including a large expansion in capabilities of the existing *Search Client* feature, after the iterative development period.

After agreed-upon prioritization of changes on the master list, researchers provided participant feedback to the Nairobi-based app developer for integration. To track and discuss progress during the 3-week period of app refinement, the app developer supplied Kisumu-based researchers with intermittent versions of mSaada, which were downloaded to study phones and reviewed for accuracy. This back-and-forth process helped facilitate effective communication between team members and allowed for successful completion of app refinement.

### Qualitative Analysis

We developed a 2-part interview guide that was used for all participants during both waves of data collection (Multimedia Appendix 1). First, we asked participants to reflect on each of the 4 features of the app. For each feature, participants were asked their opinion on usability, user control, aesthetics, comfort, ease of use, and any challenges they faced. The interview concluded with questions regarding overall impressions of the platform and thoughts about implementation and use of mSaada within a Kenyan context. We asked participants for any recommended changes or updates to the app’s layout and features, and any concerns they had about the app’s use within facilities.

We developed an initial codebook with deductive codes that mirrored the domains described above in the interview guide (usability, user control, aesthetics, comfort, and ease of use) and further refined it with inductive codes as participant feedback was gathered. We analyzed the qualitative data using thematic analysis and a 4-stage process [34]. Analysis was aided by NVivo (version 12). First, researchers reviewed all transcripts and created document memos to summarize key points from each participant and to get a strong sense of the collected data. Second, we identified deductive structural codes based on the two sections of the interview guide (ie, feature-specific feedback and overall impressions). Third, inductive thematic codes were identified via thorough review and rereview of participant transcripts. As thematic codes were identified, researchers added them to the codebook and recoded transcripts based on observed themes. Fourth, after completion of thematic coding, we wrote analytic memos for each identified theme detailing the similarities and differences in feedback between features of mSaada to broadly summarize the gathered information. Interim analysis, completed between waves 1 and 2 of data collection, used a similar process but was not aided by NVivo (version 12). To accelerate the procedure, researchers completed a consolidated version of the aforementioned 4-stage process.

### Ethics Approval

We obtained ethical approval from Duke University Campus Institutional Review Board (2019-0650) and the Kenya Medical Research Institute’s Scientific and Ethics Review Unit (2918). All participants provided written informed consent before initiation of study activities. Implementation of the study followed all local human participant research policies.

## Results

### Overview

**Figure 1:**
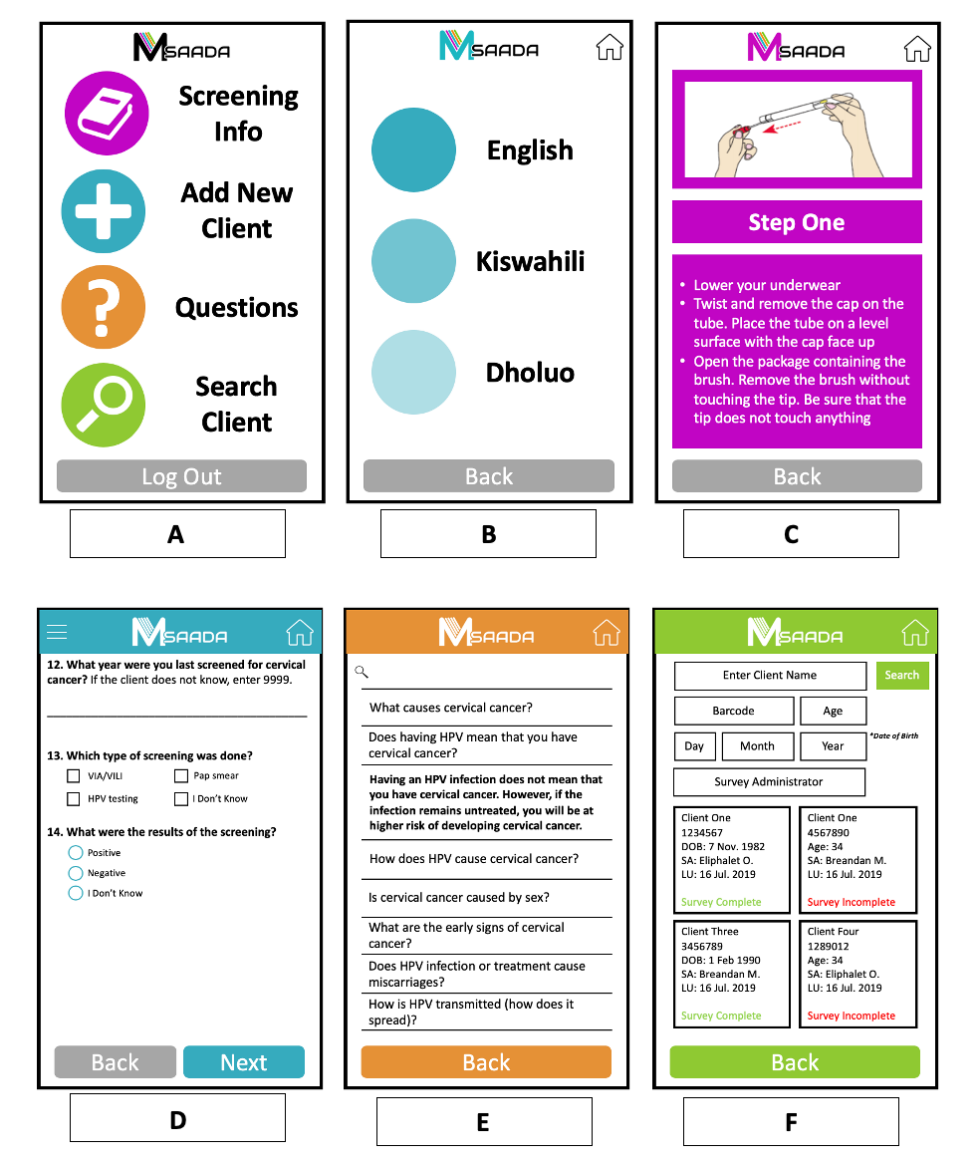
Example wireframes of mSaada: (A) home page depicting 4 main features; (B) page showing option to select language preference; (C) self-collection screening instructions within the Screening Info feature; (D) patient data entry page within the Add New Client feature; (E) searchable frequently asked questions with corresponding answer; and (F) Search Client feature illustrating client record lookup. The client-facing language used in wireframes C, D, and E is reflective of participant recommendations (see the Anticipated Workflow Considerations section). mSaada is a provider-sided app. DOB: date of birth; HPV: human papillomavirus; LU: last updated; SA: survey administrator; VIA/VILI: Visual Inspection with Acetic Acid and Lugol’s Iodine.

Feedback resulted in changes to all 4 areas of the mSaada app functionality and appearance. Changes to the app focused on improving user experience and enhancing the visual appeal of the platform. Figure 1 depicts the wireframes of the final version of mSaada after iterative development. Table 2 outlines key updates made to each feature of mSaada. Qualitative data gathered during the iterative development process were classified into four categories: ease of use, accessibility of information, anticipated workflow considerations, and acceptability.

**Table 2:** Feature-specific feedback and the resulting revisions to mSaada.

App feature and weakness identified		Changes or update made
**Add New Client**		
	Lack of clarity in questionnaire items	Refined phrasing in consultation with Kisumu-based staff
	Lack of questionnaire items gathering administrative details	Added items regarding screening location and provider name
	Questionnaire items presented only in English	Translated feature content into Swahili and Luo
**Screening Info**		
	Lack of sufficiently detailed graphics and instructions for self-collection	Updated step-by-step instructions to include more detail
	Need for additional images or graphics to aid in counseling	Included Kenya Ministry of Health Cervical Cancer Prevention Protocol for additional support
	Content presented only in English	Translated feature content into Swahili and Luo
**Questions**		
	Difficulty using built-in keyword search query	Broaden keyword search query to include synonymous terms
	Educational content presented only in English	Translated into Swahili and Luo
**Search Client**		
	Unable to effectively edit client records	Incorporated editing capabilities within feature^a^
	Search fields of database query returning broad group of client records	Added additional search fields to aid in narrowing of client records

^a^Not completed before pilot testing.

### Ease of Use

Overall, participants were comfortable using the main features of mSaada. Participants cited previous mobile phone use, specifically smartphone use, as a main reason for their comfort with the app:

The practitioners here in Kisumu, I think most of them are used to these phones, so I don’t think they will have many challenges, maybe it is just a matter of familiarizing with the app. It’s quite different, but almost everyone has smartphones, so they’re used to them.

Expert 4

When asked about their use of specific features of mSaada, participants reported ease with all features except the *Questions* feature. They liked the logical flow from screen to screen within each feature, allowing for overall easy use of the app. For example, participants liked the use of the *Back* and *Next* buttons for navigation within the app and felt the scrolling and swiping aspects of the platform were effective, responsive, and straightforward. Regarding the *Questions* feature, participants reported difficulty in identifying the correct keyword necessary to locate the needed information. When asked about their challenges with the *Questions* feature, participants believed this difficulty was due to a lack of familiarity and experience with mSaada, not a weakness in the design or functionality of the platform. Participants felt that the use of the *Questions* feature would improve with repeated exposure and did not recommend any changes:

I think the feature helps a lot in replying to questions. All we have to do is master the concept, so that during the [keyword search] you already have an insight. That way when a client asks you a question, you know what to type and then get the correct information.

End User 4

Participants concluded that the most effective way to become well versed with the app was through role-play and other simulation activities. They recommended detailed and engaging trainings for CHVs to facilitate successful use before going to the field.

### Accessibility of Information

Accessibility of information emerged as both a strength and weakness of mSaada. Overall, participants commended the use of very simple, direct, nonmedical jargon within the app. Participants suggested that this would likely increase the usefulness of mSaada, as CHVs would be able to effectively communicate necessary information with clients at a contextually appropriate level of understanding, regardless of their lack of formal medical training. In addition, the format of educational content, specifically within the Cervical Cancer Education Module, was well received, as participants found the scrollable nature of the electronic information easier to use in comparison with the bulky flip charts currently used within facilities.

Although strengths in the presentation of information were identified, all participants highly recommended translation of app content into Swahili, a national language of Kenya, as well as Luo, a local language in western Kenya, so that CHVs could effectively convey important information to clients of any language preference. It was mentioned that, by presenting only an English version of the app, CHVs were responsible for translation of information on the fly to non-English–speaking clients, which could allow for inconsistencies or inaccuracies in the transmission of information from app to CHV to client. Thus, to lower the burden of responsibility on CHVs, participants recommended that all information intended for client consumption be uniformly translated to both local languages.

In addition to the inclusion of local languages, participants highlighted sentences and phrases included within the app’s *Add New Client* and *Screening Info* features that were not well understood or did not appropriately convey the intended meaning of the statement within a Kenyan context. To ensure effective use of mSaada and reduce varied or unintended interpretation of information, participants placed emphasis on the need to assure that phrasing of statements was nuanced and reflective of the speech patterns of those in the target area.

Finally, participants were concerned with the lack of ability to access or edit completed client records once information was entered into the app. They felt it was important to include an editing aspect within the *Search Client* feature as well as a way of uniquely identifying client records to get “all of the info about [a] person.” Participants cited examples from past experience where small typos had been made or where there were questions about a certain client record that needed further evaluation.

### Anticipated Workflow Considerations

Overall, participants believed that mSaada would help accelerate the screening and client data collection processes. Errors and inefficiencies within the app were highlighted, and changes were recommended, especially within the *Add New Client* feature. Participants emphasized the need for the incorporation of clinically relevant logic checks throughout the clinical questionnaire. Recommended checks included the following: age, to ensure clients are within the recommended target age range for screening; history of hysterectomy, to ensure no contraindication of screening; and pregnancy status, which is of clinical importance during the treatment of HPV infection or cervical cancer. In addition, participants recommended refinement of response options, specifically the alphabetizing of lengthy dropdown menus, within the clinical questionnaire to make the data collection process more efficient.

Participants also emphasized the need to cater the presentation of information within mSaada toward end users (CHVs) for successful and efficient use within facilities. Participants felt that the wording of items within the clinical questionnaire should be constructed so that CHVs could read the text directly from the app as if they were talking to a client, rather than having to reframe the question after reading. During role-play activities, participants often stumbled over questionnaire items not written in this way.

Finally, participants identified a data persistence issue within the *Add New Client* feature, which they felt would negatively impact the CHV workflow within clinical settings. The possibility of data loss and the need for re-entry of client data concerned participants.

The issue...was that when you go back [to a previous screen using the “Back” button], now you have to again key in the same set of information and this might be a problem if you have a number of patients who are on the line because we may make errors and we may also want to change [answers] without doing away with the whole information.

Lay Person 2

### Acceptability

Participants believed that mSaada included all components and features necessary to aid CHVs in the successful screening of clients. They found the Cervical Cancer Education Module within the *Screening Info* feature to be comprehensive but concise. In addition, although not exhaustive, participants found the *Questions* feature content to be extremely useful for client education and believed the information covered many of the main topics about which a client might inquire. Participants did, however, recommend that the 4 main features on the home screen be reordered. Participants believed that presenting the features in chronological order of a client’s screening visit would help reduce confusion of app users (CHVs) and help facilitate the visit.

The “Add New Client” is what we’ll be using mostly for our new clients. It [“Screening Info”] should be the second because after adding a patient is when you go to the “Screening Info” and the education module. And then you go to “Questions”. And lastly the “Search Client” should be the last because maybe you could use that later, after the interview.

End User 4

Finally, participants recommended the addition of a fifth feature for administrative purposes. The additional feature, a report-generating tool, was recommended to enhance mSaada’s usefulness at the facility and health system levels. Participants cited a need for up-to-date records on clinical outcomes and services rendered, which is requested by the Ministry of Health.

## Discussion

### Principal Findings

To our knowledge, we are the first to describe the development of an app designed for health workers offering HPV-based cervical cancer screening services in Kenya. The iterative development process, whereby relevant stakeholders with a diversity of perspectives provided input in a cyclical manner, proved to be effective at creating a viable, functioning app for pilot testing. The stakeholder-engaged, iterative process, which has been used in many studies [18-20,35,36], yielded critical insight that could not have been gathered otherwise. For example, many of the word choices and sentence structures included within the platform were considered to be unclear and not likely to be understood by clients during use, even though this language originated from materials provided by Kenyan research staff. Although the app was designed to improve the quality of counseling, having end users test the app revealed additional concerns about the possibility of miscommunication. These lessons learned call to attention the need for highly engaged, locally driven processes of intervention development, even outside of mHealth approaches.

### Comparison With Prior Work

Many mHealth interventions are developed with a singular intent and, therefore, use only 1 approach to achieve their purpose, through communication, education, data collection, or information sharing [10]. This method was countered in this study, as participants applauded mSaada’s multifaceted nature, offering a comprehensive solution to many of the challenges experienced in the delivery of screening services. This stands as an example of the importance of multidimensional approaches for future mHealth interventions.

A key strength of this study is the feedback cycle. The mSaada platform underwent multiple rounds of testing and refinement in the 2-month study period, and this process involved a variety of individuals of varying engagement in cervical cancer screening. In addition, key stakeholder and end user feedback was integral to decision-making and revisions of the platform. A study by Fishbein et al [19] showed that the inclusion of a broad range of stakeholders and perspectives proved beneficial for the development of the app, and the sense of cocreation likely contributed to the resulting acceptability of the app. By striving to produce a product that was context specific and relevant to its target audience, those with the most firsthand knowledge and deepest insight into the success and failure of current screening efforts were able to drive mSaada’s development, likely resulting in a better and more useful final product. Another strength of this study was the use of qualitative methods to provide detailed feedback, opinions, and perspective on the app’s development and use. Qualitative methods, if executed well, can produce a plethora of actionable information for use in intervention development and further revision. Many usability testing frameworks emphasize the use of qualitative methods when developing and evaluating mHealth interventions [33].

### Limitations

There were also a few limitations to this study. First, although we encouraged feedback from a variety of individuals, our small sample size could have missed important feedback. Given the novel nature of the app within this setting, this study was focused on feedback from app end users. However, feedback from women being screened by providers using the app is critical to its implementation and was missing from our study. In addition, although consulted informally, hospital and district health administrators were not included within our study sample, therefore, insight into how mSaada might integrate into facilities from a macro level was not gathered. Finally, participants were given only 3 hours, on average, to use the app before providing feedback. This limited interaction may not have provided sufficient time to fully explore and identify an issue with the platform, and functionality in clinical settings should be tested in a pilot study. Future mHealth development studies should strive to gather feedback from a sufficient number of stakeholders at all levels of the health system and provide ample interaction with their proposed platforms.

### Conclusions

This study demonstrates the usefulness of iterative approaches to mHealth development. By engaging a variety of key stakeholders, we were able to quickly develop a mobile app that would be well received, have ownership among end users, and ensure readiness for small-scale pilot testing. In this study, we show a process for an iterative approach to app development that builds on context-specific preliminary work to further improve the functionality before introduction in a clinical setting.

## Appendix

Multimedia Appendix 1 In-depth interview guide.

## Abbreviations

CHV: community health volunteer
HPV: human papillomavirus
mHealth: mobile health
WHO: World Health Organization
