# Supplementary material for: Iterative Development of a Mobile Phone App to Support Community Health Volunteers During Cervical Cancer Screening in Western Kenya: Qualitative Study
Source: JMIR Form Res. 2022 Feb 24;6(2):e27501. doi: 10.2196/27501 (PMC8914757; doi:10.2196/27501)
Supplement: Multimedia Appendix 1 [file formative_v6i2e27501_app1.docx]

**Iterative Development In-depth Interview Guide**

*(For questions 1-4, we would like to understand their experience with each specific function of the app. We want them to think critically about their experience with each specific function and provide as much feedback as possible so we can work to improve mSaada and make sure it is offering all the necessary features to complete their work.)*

1. **We’d like to learn about your experience with the counseling support feature of mSaada: Cervical Cancer Education Module and Screening Images.**

- How did it function? Please describe any issues you had when using this feature, including what worked well and what did not.
- How comfortable did you feel using this feature of mSaada?
- How easy was it to use this feature? What made it easy? What were the challenges?
- What did you think about the content of the Education Module and Screening Images?
  - Prompts: Appropriate? Understandable? Helpful?
- What changes would you recommend to this feature?
- What additions would you add to this feature?
- What concerns do you have about this feature of mSaada?
- Do you have any additional feedback on this feature that was not already mentioned?

1. **We’d like to learn about your experience with the Frequently Asked Questions feature of mSaada.**

- How did it function? Please describe any issues you had when using this feature, including what worked well and what did not.
- How comfortable did you feel using this feature of mSaada?
- How easy was it to use this feature? What made it easy? What were the challenges?
- What did you think about the content of the Frequently Asked Questions?
  - Prompts: Appropriate? Understandable? Helpful?
- What changes would you recommend to this feature?
- What additions would you add to this feature?
- What concerns do you have about this feature of mSaada?
- Do you have any additional feedback on this feature that was not already mentioned?

1. **We’d like to learn about your experience with the Specimen Tracking feature of mSaada.**

**[If they did not use this function, move to the next session]**

- How did it function? Please describe any issues you had when using this feature, including what worked well and what did not.
- How comfortable did you feel using this feature of mSaada?
- How easy was it to use this feature? What made it easy? What were the challenges?
- What changes would you recommend to this feature?
- What additions would you add to this feature?
- What concerns do you have about this feature of mSaada?
- Do you have any additional feedback on this feature that was not already mentioned?

1. **We’d like to learn about your experience with the Patient Tracking feature of mSaada.**

**[If they did not use this function, move to the next session]**

- How did it function? Please describe any issues you had when using this feature, including what worked well and what did not.
- How comfortable did you feel using this feature of mSaada?
- How easy was it to use this feature? What made it easy? What were the challenges?
- What changes would you recommend to this feature?
- What additions would you add to this feature?
- What concerns do you have about this feature of mSaada?
- Do you have any additional feedback on this feature that was not already mentioned?

*(For question 5, we are interested in learning about their overall opinion of mSaada, to better understand how they perceive the platform as a whole.)*

1. **Tell me about your overall opinion of mSaada.**

- What do you think about the formatting of the app? What would you change?
- What do you think about the color scheme of mSaada? Would you recommend different colors? If so, what colors?
- Do you think any features are missing from mSaada? If so, what features?
- Do you think any current features should not be in mSaada? If so, which ones?
- How easy was it for you to learn how to use mSaada? What would make it easier for you and other Community health Volunteers to learn?
- What was your least favorite aspect of mSaada?
- What was your favorite aspect of mSaada?
